# Supplementary material for: Viral Community and Novel Viral Genomes Associated with the Sugarcane Weevil, Sphenophorus levis (Coleoptera: Curculionidae) in Brazil
Source: Viruses. 2025 Sep 28;17(10):1312. doi: 10.3390/v17101312 (PMC12567637; doi:10.3390/v17101312)

**Supplementary Figure 1: Rarefaction curves of viral communities at the family level (mean ± SE), classified using RAT**

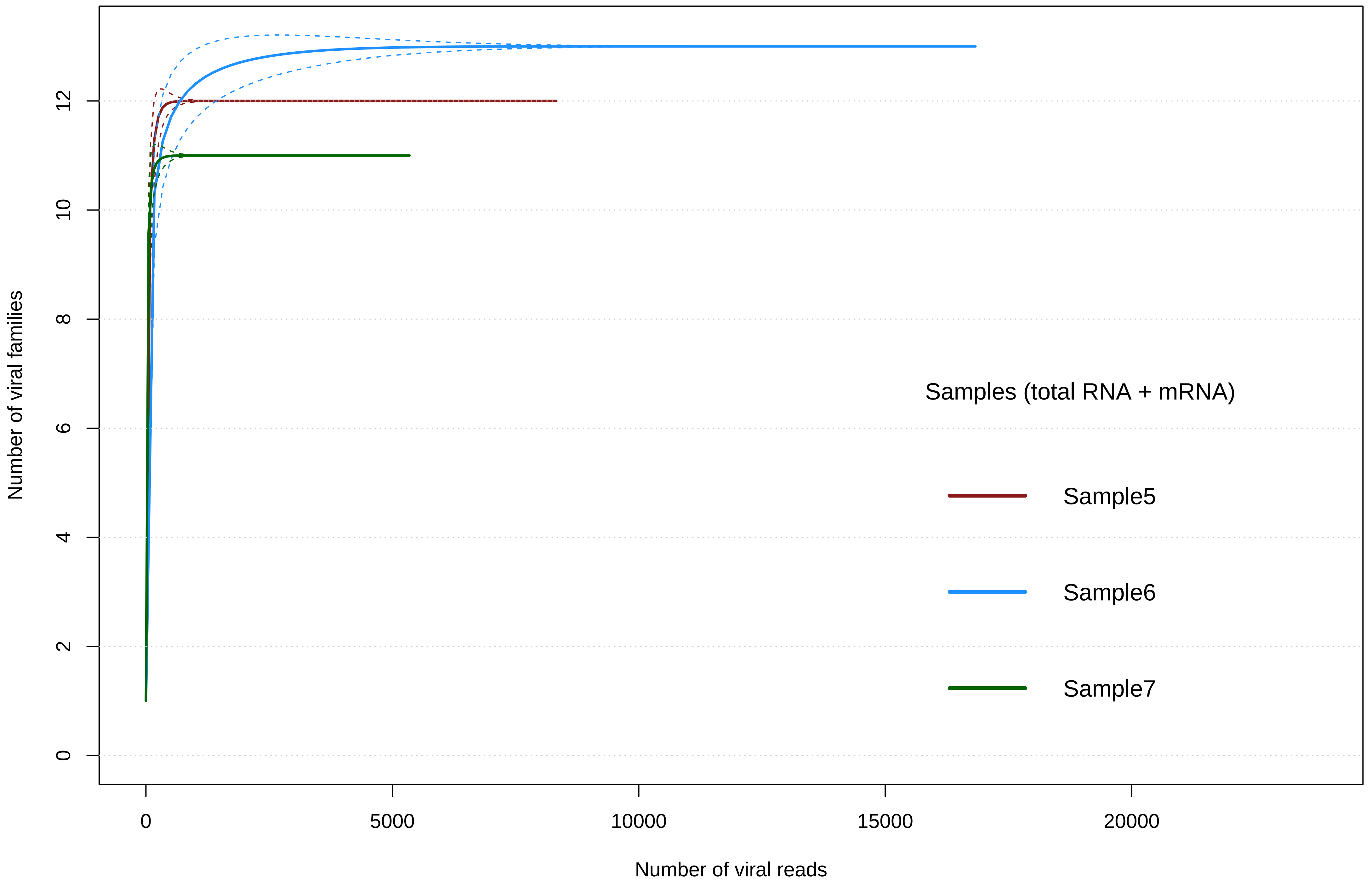

Supplement: Supplementary file 1 [file viruses-17-01312-s001.zip › Supplementary Figure S1.pdf]
