# Supplementary material for: Viral Community and Novel Viral Genomes Associated with the Sugarcane Weevil, Sphenophorus levis (Coleoptera: Curculionidae) in Brazil
Source: Viruses. 2025 Sep 28;17(10):1312. doi: 10.3390/v17101312 (PMC12567637; doi:10.3390/v17101312)
Supplement: Supplementary file 1 [file viruses-17-01312-s001.zip › Supplementary Table S1.pdf]

Supplementary Table S1. Reads assigned by RAT to RNA and DNA virus families, genus or species based on highest similarity.

	Number of reads						
	Sample 5		Sample 6		Sample 7		
**Classification**	**PolyA**	**Total RNA**	**PolyA**	**Total RNA**	**PolyA**	**Total RNA**	**Total**
**RNA**	**31036**	**287108**	**19623**	**326905**	**29263**	**269817**	**963752**
Partitiviridae		**119**	**26**	**11419**			**11564**
Drosophila biauraria male killing partitivirus 1			26	11419			11445
Orestiada partiti-like virus		119					119
Totiviridae		**65**		**60**		**162**	**287**
family *Totiviridae*		10		9		23	42
Totiviridae sp.		55		51		139	245
Chuviridae		**2161**		**864**		**1583**	**4608**
Coleopteran chu-related virus OKIAV151		2161		864		1583	4608
Aliusviridae		**287**		**271**		**403**	**961**
Ollusvirus coleopteri		287		271		403	961
Phasmaviridae		**555**		**1241**		**310**	**2106**
Coleopteran phasma-related virus OKIAV236		249		208		310	767
Orthophasmavirus		306		1033			1339
Orthomyxoviridae	**11**	**409**		**348**		**511**	**1279**
Coleopteran orthomyxo-related virus OKIAV200	11	389		273		464	1137
Hymenopteran orthomyxo-related virus OKIAV174						9	9
family *Orthomyxoviridae*		20		75		38	133
Rhabdoviridae		**121**		**203**		**256**	**580**
Coleopteran rhabdo-related virus OKIAV200		95		203		103	401
Ledantevirus barur		10					10
Ledantevirus oita						22	22
Lepidopteran rhabdo-related virus OKIAV11						12	12
Tupavirus durham		6					6
family *Rhabdoviridae*		10				119	129
Tombusviridae						**49**	**49**
Coleopteran tombus-related virus						49	49
Virgaviridae				**31**			**31**
Thrips tabaci associated virga-like virus 2				31			31
Benyviridae				**11**			**11**
**NA**	**31025**	**283391**	**19597**	**312457**	**29263**	**266543**	**942276**
Bercke-Baary *Melophagus* reo-like virus		157117		175534		104402	437053
Chaq virus				189			189
Diabrotica undecimpunctata virus 2				8		19	27
Gonipterus platensis bunyan-like virus	1025	501	556	393	294		2769
Hypera postica associated virus 1	30000	65648	18946	28654	28861	66987	239096
Riboviria sp.		60125	95	107679	108	95135	263142
**DNA**	**672**	**4389**	**513**	**2448**	**326**	**4711**	**13059**
Polydnaviriformidae	**628**	**2099**	**431**	**821**	**263**	**1177**	**5419**
Bracoviriform facetosae	628	2099	431	821	263	1126	5368
Ichnoviriform fumiferanae						51	51

Adintoviridae	**26**	**1463**	**34**	**444**	**51**	**345**	**2363**
Bos-associated insect adintovirus 2	26	7	34	117	51		235
Drosophila-associated adintovirus 2		1439		327		307	2073
Megastigmus wasp adintovirus		8				38	46
family *Eupolintoviridae*		9					9
Iridoviridae		**82**		**117**		**149**	**348**
Invertebrate iridescent virus 31		82		117		149	348
Nudiviridae		**121**					**121**
Alphanudivirus tertidromelanogasteris		121					121
Parvoviridae		**219**		**639**		**123**	**981**
Dendrocopos leucotos parvoviridae sp.				39			39
family *Parvoviridae*		219		600		123	942
**NA**	**18**	**405**	**48**	**427**	**12**	**2917**	**3827**
Drosophila-associated filamentous virus				97			97
Leptopilina boulardi filamentous virus		227		100		340	667
Microctonus hyperodae filamentous virus	18	178	48	230	12	2577	3063
**Total**	**31708**	**291497**	**20136**	**329353**	**29589**	**274528**	**976811**

NA = not assigned
